# Health literacy: Prevalence and determinants in Lagos State, Nigeria

**DOI:** 10.1371/journal.pone.0237813

**Published:** 2020-08-13

**Authors:** Yetunde Abiola Kuyinu, Toriola Temitope Femi-Adebayo, Bisola Ibironke Adebayo, Ibipo Abdurraheem-Salami, Olumuyiwa Omotola Odusanya

**Affiliations:** 1 Department of Community Health and Primary Healthcare, Lagos State University Teaching Hospital, Ikeja, Nigeria; 2 Department of Community Health and Primary Healthcare, Lagos State University College of Medicine, Ikeja, Nigeria; Universita degli Studi della Campania Luigi Vanvitelli, ITALY

## Abstract

**Background:**

Poor health literacy has been associated with poorer physical and mental health function, and higher emergency department and hospital utilizations. The study was conducted to measure the prevalence of health literacy and its determinants among Lagos State residents.

**Methods:**

A descriptive cross-sectional study was conducted in three local government areas in Lagos State. Health literacy was assessed using the Brief Health Literacy Screening tool (BHLS), a three-item tool with possible scores ranging between 3 and 15. Health literacy was classified as inadequate (≤ 9) or adequate (>9).

**Results:**

A total of 1831 respondents participated in the study, among whom, 952 (52%) were women. The mean age of respondents was 31.7 (±10.5) years. Three-quarters (74.8%) of respondents had adequate health literacy. Adequate levels of health literacy were associated with being female (OR, 1.35; 95% CI, 1.07–1.71), frequent use of the broad cast media as source of information (OR, 1.33; 95% CI, 1.03–1.70), frequent use of the internet as source of information (OR, 1.49; 95% CI, 1.13–1.96). Adequate health literacy was also associated with having knowledge of a frequently prescribed antibiotic (OR, 1.67; 95% CI, 1.32–2.12) and being more comfortable with the use of the English language (OR, 1.71; 95% CI, 1.32–2.22).

**Conclusion:**

Gender, the use of broadcast media and the internet are predictive of adequate health literacy and should be taken into consideration in planning health interventions.

## Introduction

Health literacy refers to the degree to which individuals have the capacity to obtain process and understand basic health information and services necessary to take appropriate health decisions [1]. The World Health Organization (WHO) defines it as the cognitive and social skills which determine the motivation and ability of individuals to gain access to, understand and use information in ways which promote and maintain good health [2]. Heath literacy means more than being able to read pamphlets, it includes the totality of processes involved in appreciating and utilizing health information effectively and is essential if patients are to exercise control over their own health. It has been classified into three levels; basic or functional health literacy, communicative or interactive health literacy and critical health literacy [3,4]. Functional health literacy has been described as a fundamental requirement for effective engagement of patients with health-related decision-making [3]. A systematic review of health literacy and health outcomes reported that patients with low levels of literacy were generally one and half to three times more likely to experience a given poor health outcome [5]. Poor levels of health literacy have been associated with more hospitalizations, greater use of emergency care and poorer ability to interpret drug labels, health messages and higher mortality rates among elderly persons [6]. Health literacy has also been found to be associated with medication adherence; a study conducted in Heris County, Iran reported a considerable association between health literacy and self-reported medication adherence among patients with primary hypertension [7]. A study amongst Medicare-managed enrolees in the United States of America (USA) reported that inadequate health literacy independently predicted all cause mortality and cardiovascular death among community-dwelling elderly persons [8]. In another study, the weighted prevalence of inadequate health literacy was 26% and was associated with level of education, ethnicity and age but not gender [9]. Amongst patients with type 2 diabetes mellitus in the USA, the prevalence of inadequate functional health literacy was 29% and was significantly associated with educational level below a high school education [10].

The problem of low health literacy is global. Approximately 21% of American adults are functionally illiterate, and another 27% have marginal literacy skills yet many physicians do not recognize the problem or have the confidence to deal with it [11]. About one in ten persons in Europe have inadequate health literacy [12]. In Ireland at least one in seven adults were found to have limited health literacy and the patients with limited health literacy were significantly more likely to report problems with using health materials received from a doctor or pharmacist [13], while a systematic review and meta-analysis conducted in Iran revealed that health literacy in the Iranian population was inadequate and borderline [14]. Also the prevalence of low health literacy in China was 85% and was significantly associated with level of education, occupation, annual household income but not with gender or age [15].

In Nigeria, only 38% of in-school adolescents possessed adequate health literacy skills and there was a significant relationship between health literacy skills level and internet health information use [16]. Furthermore, in Nigeria, low level of health literacy was found to be associated with poor medication adherence [17]. A review paper identified causes of low levels of health literacy in Nigeria to include culture and belief system, low educational level, low socio-economic status and ineffective communication [18]. The poor health outcomes and late presentation to the health system by Nigerians in part may be due to inadequate health literacy [19,20]. Without investigating the subject of health literacy and its determinants, desired improvements in the health status of citizens may remain a mirage as they will only be treated in the hospitals and clinics and be sent back to face the same factors and circumstances that contributed to their ill health. Therefore this study was conducted to determine the prevalence and determinants of health literacy among the adult general population resident in Lagos State, southwest Nigeria with a view to providing useful information to health workers and policy makers.

## Materials and methods

### Background information to study area

The study took place in Lagos State, the commercial capital of Nigeria and the second-largest state population-wise. Lagos State is located in South-western Nigeria. It has three senatorial districts comprising 20 Local Government Areas (LGAs) in all. The state is cosmopolitan in nature and can be referred to as “mini Nigeria” because all the three major ethnic groups; Yoruba, Hausa and Igbo domicile in Lagos. However, the official language is English, medical care is often provided in English Language and drugs prescribing are also in English. Lagos State has a population growth rate of 600,000 per annum and a population density of 4193 persons per square kilometre [21].

### Study design

A cross-sectional study was conducted among adults (older than 18 years) living in Lagos State. The study took place over a three-month period, between October and December 2018.

### Sample size determination

The statistical assumptions used to determine the sample size for the study applying the Fisher’s formula, were a standard normal deviate at 95% confidence interval (1.96), prevalence rate (proportion of individuals with poor health literacy) of 0.47 [22], precision of ± 3%, a 10% non-response rate and effect size of 1.5. Thus the minimum sample size for the study was 1,772.

### Sampling techniques

Participants were selected using a multi-stage sampling technique. In the first stage, two urban (Ikeja and Alimosho) and one rural (Epe) LGAs were randomly selected by balloting from the list of already existing urban (16) and rural (4) LGAs in Lagos State. In the second stage, three wards were selected by simple random sampling by balloting from the selected LGAs. In the third stage, using a sampling frame of all the streets in the selected wards, a minimum of ten streets were selected by using a table of random numbers. The fourth stage involved the selection of houses on each street by systematic random sampling. The index house was determined by picking an integer between one and the sample interval by balloting. The subsequent houses were then determined by systematic random sampling based on the calculated sample intervals. In the fifth stage, one household was selected by balloting and a consenting adult was approached for the study. Where there was more than one consenting adult, one was chosen by balloting. Thirty respondents were selected from each street and equal numbers of respondents were selected from each LGA.

### Study instrument

Health literacy was measured using the Brief Health Literacy Screen (BHLS). The choice of the BLHS was because it is easy to use and its focus that goes beyond the ability to read. It measures function, self-efficiency and need for assistance. The BHLS instrument measures health literacy using three items; "How often do you have someone help you read hospital materials?" "How confident are you filling out medical forms by yourself?" and "How often do you have problems learning about your medical condition because of difficulty understanding written information?" A 5-point Likert-type scale was used for scoring each of the items. The BHLS score is based on the sum of the three non-weighted items and can range from 3 to 15, with a higher score indicating higher health literacy. It takes about 1.5 minutes to fill. The tool was developed as a shorter tool than other tools such as the Short Test of Functional Health Literacy (STOFHLA) and Rapid Estimate of Adult Literacy (REALM) [23]. It has been shown to have a significant association with these two instruments and accurately identified participants with inadequate health literacy skills [23]. This instrument has been validated by previous study to test functional health literacy in adults, the area under the receiver operating characteristic curve was found to between 0.76 and 0.87 for the three items [24]. Face validity of the instrument was done by the authors reviewing the tool by consensus, and ensuring it met the objectives of the study. The instrument was pretested in another LGA (Mushin LGA) among 50 residents and was found to be usable in our environment. The reliability of the BHLS instrument using the Cronbach’s coefficient alpha was 0.85.

### Data collection

A semi-structured interviewer-administered questionnaire was administered to respondents using a Kobo Collect form in English language. The questionnaire included information on socio-demographic characteristics of participants, health-seeking behaviour, languages spoken and sources of health information. In addition, questions about complications of use of acetaminophen (paracetamol, a commonly used over-the counter analgesic) and dosage of ampicillin-cloxacillin (a commonly prescribed antibiotic that is available even as an over-the- counter in Nigeria) were asked. Two research assistants who were university graduates were trained on data collection and research ethics obtained data for the study.

### Data analysis

Univariate analysis was done to generate frequencies, means and standard deviation. Exposure to sources of information was graded as frequent, if greater than three days in a week and infrequent if less. Health literacy was classified as inadequate (≤ 9) or adequate (>9) [25]. The Chi-square test was used to measure the association between health literacy level and respondents’ characteristics. Logistic regression was used to identify predictors of adequate health literacy found significant at the bivariate analysis. The level of statistical significance was set at p ≤ 0.05.

### Ethical considerations

Ethical clearance for the study was obtained from the Lagos State University Teaching Hospital Health Research Ethics Committee with Reference Number LREC.06/10/1056. The participants were informed of the objectives of the study and its potential benefits for the health system and the State. The risk of harm was minimal as there were no invasive procedures. Written informed consent was obtained from each participant prior to enrolment in the study.

## Results

### Socio-demographic characteristics of respondents

A total of 1,831 respondents were recruited into the study and slightly above half (52%) were females. The mean age of respondents was 31.7±10.5 years. This result is comparable to the demographic characteristics of Lagos State. Up to 39.9% of respondents reported having completed secondary school and only about a third had a first degree from a university or a higher national diploma from polytechnics. The most frequent source of information was the internet (72%). With regards to literacy of English and other languages, 92% could read the English language compared with the 72% that could read a Nigerian language. Slightly over half (55%) of respondents were more comfortable with the English language in contrast with a Nigerian language (Table 1).

**Table 1:** Socio-demographic characteristics of respondents.

Socio-demographic Characteristics	Frequency	Percentage
**Sex**		
Male	885	48.3
Female	946	51.7
**Age-group**		
≤ 29 years	884	48.3
30–39 years	596	32.6
40–49 years	206	11.3
50–59 years	96	5.2
≥ 60 years	49	2.7
**Highest Educational Level**		
No formal education	21	1.1
Primary education	77	4.2
Secondary/technical	731	39.9
OND	289	15.8
HND/Bachelor’s degree	634	34.6
Post-graduate degree	79	4.3
**Income category**		
≤ N5,000	104	5.7
N5,001—N20,000	469	25.6
N20,001—N50,000	704	38.4
N50,001—N100,000	464	25.3
N100,001—N200,000	55	3.0
≥N200,001	35	1.9
**Frequent sources of information**		
Print media	509	27.8
Broadcast media	1143	62.4
Internet	1320	72.1
**Good ability to read a language**		
English Language	1681	91.8
Nigerian Language(n = 1672)	1195	71.5
**Language most comfortable with**		
English Language	1021	55.8
Nigerian Language	810	44.2

**Abbreviations**: OND = Ordinary national diploma, HND = Higher national diploma

### Health literacy assessment

Just about half of the respondents claimed they never needed someone else’s help in reading hospital material, while 55% had some problems learning about their medical conditions because of difficulties in understanding written information. About 50% of the respondents reported they were always confident of filling medical forms. Three-quarters of the respondents (74.8%) had adequate levels of health literacy (Table 2).

**Table 2:** Assessment of health literacy amongst respondents.

Variable	Frequency (n = 1831)	Percentage
**Frequency in requiring someone’s help in reading hospital materials**		
Never	918	50.1
Occasionally	455	24.8
Sometimes	149	8.1
Often	151	8.2
Always	158	8.5
**Occurrence of problems learning about medical condition because of difficulty understanding written information**		
Never	829	45.3
Occasionally	358	19.6
Sometimes	220	12.0
Often	168	9.2
Always	256	14.0
**Confidence in filling out medical forms by oneself**		
Always	889	48.6
Often	404	22.1
Sometimes	242	13.2
Occasionally	132	7.2
Never	164	9.0
**Overall health literacy**		
Adequate (score >9)	1370	74.8
Inadequate (score≤ 9)	461	25.2

### Factors associated with health literacy

Factors associated with health literacy on bivariate analysis are shown on Table 3. Female respondents had a significantly higher health literacy level compared to male respondents. Age and educational level were also significantly associated with health literacy. However, there was no association between income and health literacy. Respondents with secondary and tertiary levels of education were more likely to have adequate health literacy compared with those with only primary education or less. Significant associations were found with correct knowledge of the number of ampicillin-cloxacillin capsules to be taken and knowledge of Nigerian and English languages (in spoken and written forms) and adequate health literacy. Having been diagnosed with a chronic disease was not associated with adequate health literacy levels. Reading of medication leaflet inserts before use of medicines was not significantly associated with health literacy.

**Table 3:** Factors associated with health literacy amongst respondents.

Variables	Adequate health literacy	Inadequate health literacy	OR (95% CI)	Chi-square	p-value
**Sex**					
Female	632 (71.4)	253 (28.6)	1.42 (1.15–1.75)	10.57	0.001
Male	738 (78.0)	208 (22.0)			
**Age**					
< 40 years	1142 (77.2)	338 (22.8)			
≥ 40years	228 (65.0)	123 (35.0)	1.82 (1.4–2.34)	22.4	<0.001
**Educational level**					
Primary or none (ref)	49 (50.0)	49 (50.0)			
Secondary	522 (71.4)	209 (28.6)	2.50 (1.63–3.82)	49.43	<0.01
Tertiary	799 (79.7)	203 (20.3)	3.94 (2.57–6.02)		
**Income**					
≤ N50,000	963 (75.4)	314 (24.6)	1.11 (0.88–1.39)	0.78	0.38
> N50,000	407 (73.5)	147 (26.5)			
**Clinical diagnosis with a disease**					
Yes	25 (26.30)	70 (73.7)	1.06 (0.67–1.70)	0.07	0.79
No	436 (25.1)	1300 (74.9)			
**Information sources**					
Print media					
Infrequent	1014 (76.7)	308 (23.3)			
Frequent	356 (69.9)	153 (30.1)	1.41 (1.13–1.78)	8.92	0.003
Broadcast media					
Infrequent	496 (72.1)	192 (27.9)			
Frequent	874 (76.5)	269 (23.5)	0.79 (0.64–0.99)	4.56	0.037
Internet					
Infrequent	327 (64.0)	184 (36.0)			
Frequent	1043 (79.0)	277 (21.0)	0.47 (0.38–0.59)	44.13	<0.001
**Aware of complications of Paracetamol**					
Yes	209 (70.8)	86 (29.2)	1.27 (0.97–1.68)	2.95	0.086
No	1161 (75.6)	375 (24.4)			
**Have read leaflet inserts of medication before use**					
Yes	474 (73.8)	168 (26.2)	0.92 (0.74–1.15)	0.43	0.51
No	896 (75.4)	293 (24.6)			
**Aware of number of Ampicillin/ Cloxacillin capsules given per dose to an adult**					
Yes	922 (80.1)	229 (19.9)	2.08 (1.68–2.58)	45.14	<0.001
No	448 (65.9)	232 (34.1)			
**Aware of number of times per day, Ampicillin /Cloxacillin is to be taken**					
Yes	13 (65.9)	7 (35.0)	0.62 (0.24–1.66)	0.57	0.45
No	1357 (74.9)	454 (25.1)			
**Number of languages spoken**					
One	296 (64.8)	161 (35.2)	1.95 (1.55–2.45)	32.67	<0.001
Two or more spoken	1074 (78.2)	300 (21.8)			
**Ability to read English**					
Good	1288 (76.6)	393 (23.4)	2.72 (1.93–3.82)	35.23	<0.001
Poor	82 (54.7)	68 (45.3)			
**Ability to write English**					
Good	1278 (76.5)	392 (23.5)	2.44 (1.75–3.41)	29.29	<0.001
Poor	92 (57.1)	69 (42.9)			
**Ability to read a Nigerian language (n = 1672)**					
Good	950 (77.1)	282 (22.9)	1.49 (1.17–1.90)	10.52	0.01
Poor	305 (69.3)	135 (30.7)			
**Ability to write a Nigerian language (n = 1672)**					
Good	917 (76.7)	278 (23.3)	1.36 (1.07–1.72)	6.29	0.012
Poor	338 (70.9)	139 (29.1)			
**Language most comfortable with**					
English	835 (81.8)	186 (18.2)	2.31 (1.86–2.86)	59.35	< 0.001
Local Nigerian Language	535 (66.0)	275 (34.0)			

**Abbreviations**: OR = odds ratio, CI = confidence interval

The predictors of adequate health literacy are shown on Table 4. Women were 1.35 times more likely to have adequate health literacy compared with men [AOR: 1.35, 95% CI: (1.07–1.71)]. Those who got information from broadcast media showed higher odds of having adequate health literacy [AOR: 1.33, 95% CI: (1.03–1.70)]; as well as those who got information from the internet [AOR: 1.49, 95% CI: (1.13–1.96)]. Conversely, those who frequently got information from newspapers were significantly less likely to have adequate health literacy [AOR: 0.67, 95% CI: (0.51–0.88)]

**Table 4:** Predictors of Health Literacy amongst respondents.

Variable	Adequate Health literacy n (%)	Inadequate health literacy n (%)	Adjusted odds ratio (95% CI)	p-value
Sex				
Male (ref)	632 (71.4)	253 (28.6)		
Female	738 (78.0)	208 (22.0)	1.35 (1.07–1.71)	0.013*
Age-group				
< 40 years (ref)	1142 (77.2)	338 (22.8)		
≥ 40 years	228 (65.0)	123 (35.0)	0.82 (0.61–1.11)	0.198
Educational level				
Primary (ref)	49 (50.0)	49 (50.0)		
Secondary	522 (71.4)	209 (28.6)	1.30 (0.76–2.19)	0.317
Tertiary	799 (79.7)	203 (20.3)	1.72 (1.00–2.97)	0.052
Gets information from newspapers				
Rarely (ref)	1014 (76.7)	308 (23.3)		
Frequent	356 (69.9)	153 (30.1)	0.67 (0.51–0.88)	0.004*
Gets information from broadcast media				
Rarely (ref)	496 (72.1)	192 (27.9)		
Frequent	874 (76.5)	269 (23.5)	1.33 (1.03–1.70)	**0.025**
Gets information the internet				
Rarely (ref)	327 (64.0)	184 (36.0)		
Frequent	1043 (79.0)	277 (21.0)	1.49 (1.13–1.96)	0.004*
Languages spoken				
Speaks one language (ref)	296 (64.8)	161 (35.2)		
At least two spoken	1074 (78.2)	300 (21.8)	1.39 (1.05–1.85)	0.022*
Self-reported ability to read English				
Poor (ref)	82 (54.7)	68 (45.3)		
Good	1288 (76.6)	393 (23.4)	1.63 (0.55–4.90)	0.378
Self-reported ability to write in				
English				
Poor (ref)	92 (57.1)	69 (42.9)		
Good	1278 (76.5)	392 (23.5)	0.84 (0.29–2.48)	0.755
Self-reported ability to read a Nigerian language				
Poor (ref)	305 (69.3)	135 (30.7)		
Good	950 (77.1)	282 (22.9)	1.88 (0.76–4.65)	0.170
Self-reported ability to write in a Nigerian language				
Poor (ref)	338 (70.9)	139 (29.1)		
Good	917 (76.7)	278 (23.3)	0.69 (0.28–1.69)	0.417
Language most comfortable with				
Local Nigerian language (ref)	535 (66.0)	275 (34.0)		
English	835 (81.8)	186 (18.2)	1.71(1.32–2.22)	<0.001*
Aware of adult dosage of ampicillin/cloxacillin				
No (ref)	448 (65.9)	232 (34.1)		
Yes	922 (80.1)	229 (19.9)	1.67 (1.32–2.12)	<0.001*

*statistically significant;

**Abbreviation**: ref = reference

With regards to self-reported reading and writing abilities, those who spoke two languages or more had higher odds of having adequate health literacy compared with those who spoke only one language [AOR: 1.39, 95% CI: (1.07–1.71)]. In addition, those who reported being more comfortable with English compared to a local Nigerian language were 1.71 times more likely to have adequate health literacy [AOR: 1.71, 95% CI: (1.32–2.22)]. Those who were aware of the right adult dose for ampicillin/cloxacillin were 1.67 times more likely to have adequate health literacy [AOR: 1.33, 95% CI: (1.32–2.12)] but higher level of education, age, self-reported ability to read and write the English language or a Nigerian language were not predictive of adequate levels of health literacy.

## Discussion

This study set out to determine the level of health literacy and its determinants in Lagos State. To the authors’ knowledge, this study may be the first time that the BHLS tool was applied in the community and to assess health literacy in the general population.

Three-quarters of the respondents had adequate levels of health literacy which was slightly lower than the 80% reported in an ambulatory facility setting in USA [22], and which is generally higher than rates seen in China [15] but comparable with rates in the USA [11], and Europe [12]. The relatively high health literacy rate may be reflective of the high adult literacy rate in Lagos State which stands at 94% for men and 85% for women [26]. However, in two of the three constructs of assessment, less than half of the respondents were fully confident of filling out medical forms by themselves or never having problems with understanding written information. This presents a challenge for their wellbeing as it could affect their ability to recognize and report medical issues, including adherence and compliance with medical instructions given by providers.

Furthermore, in this study, women had a significantly higher levels of health literacy compared to male respondents. This was similar to a study conducted in Korea, where women had a significantly higher level of health literacy than men in understanding medical forms, directions on medication bottles, and written information offered by health care providers [27]. The higher level of adequate health literacy in women could be because women generally utilize health facilities more frequently than men and as a result, may have greater exposure to medical knowledge. However, this result was different from a study conducted in Iran which reported that women were better than men in health knowledge but the men had higher mean scores for health literacy than women [7]. In addition broadcast media and the use of the internet as sources of information were predictive of health literacy as opposed to the use of the print media. This is in agreement with a Nigerian study [16]. This may indicate that the population is moving away from the print media perhaps on account of portability and cost. It may indicate that electronic media may be one -way to reach especially the younger age groups if health literacy is to improve. It must also be pointed out that accuracy of information from the internet is not always reliable thus there are gaps which trusted health authorities can fill.

This study is at variance with other studies that showed that that being of older age-groups is associated with lower health literacy scores [9,28,29], although some other studies have reported similar findings to this present study [15] indicating that the true association with age is dependent on the population studied. This lack of concordance may be because the population in this study was younger and or had more exposure to health information more than the populations in those studies where older age was found to be associated with lower levels of health literacy [28,29]. The study did not find a significant predictive association between educational level and health literacy in contrast to studies in China [30] and Spain [31] that found that health literacy increased with higher educational levels. This may be due in part to the other unidentified factors that were associated with educational level in our study. This present study found that those with better knowledge of a common antibiotic had better health literacy scores. This may be related to greater exposure to medical information from various sources and may serve as a proxy indicator for adequacy of health literacy Also being comfortable with the English language than a Nigerian language was predictive of adequate health literacy. This may be due to the fact that English language is official language and mode of communication in our health institution and most medical instructions and medication dosages were always in English Language.

We recommend that to improve health literacy, attention should be paid to providing information more through electronic and social media and that messages be targeted at the people with lower levels of education and older people as they are at more risk of inadequate heath literacy. Inter-sectoral collaboration between health and other sectors such as information, communication, information technology and education should be strengthened. While health workers may be content experts, these other groups are experts in delivery and marketing which are essential for the messages to make the desired impacts. messages. There is the need to strengthen communication in Nigerian languages even in cosmopolitan cities like Lagos as this will help improve health literacy.

The strength of our study includes a large sample size, drawn from three LGAs from each of the three senatorial districts in Lagos state, thus supporting generalizability to the population of Lagos State. However, the study also had some limitations as the BHLS tool was originally validated in clinical settings and not in the community. The tool is also self-reported and may be subject to misclassification as well as social desirability bias.

## Conclusion

Three out of four participants were classified as having adequate health literacy. Gender and source of information are important predictors of adequate health literacy and should be considered when designing interventions. Further studies are also needed to identify other determinants of health literacy in the Nigerian community.

## Supporting information

S1 File (PDF)
