# Supplementary material for: Health literacy: Prevalence and determinants in Lagos State, Nigeria
Source: PLoS One. 2020 Aug 13;15(8):e0237813. doi: 10.1371/journal.pone.0237813 (PMC7425911; doi:10.1371/journal.pone.0237813)
Supplement: S1 File — (PDF) [file pone.0237813.s001.pdf]

A MEASUREMENT OF HEALTH LITERACY AMONG LAGOS STATE RESIDENTS

Age (Last birthday)

Sex

- ◯ Male
- ◯ Female

What language did you first learn to read and write?

- ◯ English
- ◯ French
- ◯ Mother tongue
- ◯ Any other language

Languages spoken list all

- ☐ Yoruba
- ☐ Igbo
- ☐ Hausa
- ☐ English
- ☐ French
- ☐ List any other

Specify the any other

Language you are most comfortable reading and speaking

- English
- French
- Yoruba
- Hausa
- Igbo
- Yoruba
- Another local Nig Lang
- others

Highest level of education completed

- No formal education
- Primary
- Secondary/Technical college
- OND
- HND/Bachelor
- Postgraduate

With regard to languages you understand, Would you say?

With regard to English, how well do you understand it when it is spoken to you?

- Very well
- Well
- Not well
- Not at all

With regard to English, how well do you speak it?

- Very well
- Well
- Not well
- Not at all

With regard to English, how well can you read it?

- ◯ Very well
- ◯ Well
- ◯ Not well
- ◯ Not at all

With regard to English, how well can you write it?

- ◯ Very well
- ◯ Well
- ◯ Not well
- ◯ Not at all

Do you speak a local (nigerian) language

- ◯ yes
- ◯ no

If yes, specify which

____

If yes, concerning , how would you say you understand it when it is spoken to you?

- ◯ very well
- ◯ well
- ◯ not well
- ◯ not at all

If yes, concerning , how well would you say that you speak it?

- ◯ very well
- ◯ well
- ◯ not well
- ◯ not at all

If yes, concerning , how well would you say that you read it?

- very well
- well
- not well
- not at all

If yes, concerning , how well would you say that you write it?

- very well
- well
- not well
- not at all

Current employment status

- self-employed
- unemployed
- in full-time employment
- Contract/part time employment

What is your occupation

- Professional/High scale trading
- Senior Govt employee
- Clergy, High scale farming, junior govt employee, middle scale trading, teachers, technicians (medical technicians, factory technicians)
- Artisans, security man/personnel, security agent
- Labourer, messanger,apprentices, student, peasant farmer, unemployed

Monthly income (Gross)

Please select the income category

- ◯ less than 5999 per month
- ◯ 6,000-2,0999
- ◯ 21,000-50,999
- ◯ 51,999-100,999
- ◯ 101,999-200,999
- ◯ 201,000 and above

Rate your health on a scale of 1-10

- ◯ 0- Worst possible health (as bad or worse than been dead)
- ◯ 1
- ◯ 2
- ◯ 3
- ◯ 4
- ◯ 5- Half-way between worst and best state of health
- ◯ 6
- ◯ 7
- ◯ 8
- ◯ 9
- ◯ 10-Best possible health

Can you tell me how much information about current events, public affairs, and government do you get from: NEWSPAPERS

- ◯ A lot
- ◯ Some
- ◯ A little
- ◯ None

Can you tell me how much information about current events, public affairs, and government do you get from: MAGAZINES

- ◯ A lot
- ◯ Some
- ◯ A little
- ◯ None

Can you tell me how much information about current events, public affairs, and government do you get from: INTERNET

- ◯ A lot
- ◯ Some
- ◯ A little
- ◯ None

Can you tell me how much information about current events, public affairs, and government do you get from: RADIO

- ◯ A lot
- ◯ Some
- ◯ A little
- ◯ None

Can you tell me how much information about current events, public affairs, and government do you get from: BOOKS

- ◯ A lot
- ◯ Some
- ◯ A little
- ◯ None

Can you tell me how much information about current events, public affairs, and government do you get from: FAMILY & FRIENDS

- ◯ A lot
- ◯ Some
- ◯ A little
- ◯ None

Where do you mostly go if you are sick, or to treat a general health problem

- ◯ Private clinic/hospital
- ◯ Government clinic or Hospital
- ◯ Traditional or Homeopathic healer
- ◯ Clinic run by a non governmental organisation or church
- ◯ other

Have you used any health facility in the last 6 months

- yes
- no

If yes to the above, which type of facility did you visit?

- private hospital
- Government clinic or hospital
- traditional/homeopathic healer
- clinic by a NGO/church
- other

please specify others

How often do you generally seek health care at a clinic or hospital (check one)

- Twice a year or more
- Once per year
- Less than once a year but at least twice in past 5 years
- Once in the last 5 years
- Never in past 5 years
- Other

Have you been diagnosed with any chronic health condition such as hypertension, Diabetes, Cancer, Joint disease, skin disease, organ failure.

- yes
- no

If yes, please specify

- [ ] Hypertension
- [ ] DM
- [ ] Sickle cell disease
- [ ] Asthma
- [ ] Tuberculosis
- [ ] Others, please specify

Are you presently on any medication?

- yes
- no

If yes, specify

____

. Have you ever used one or more of the following tobacco products (cigarettes, snuff, chewing tobacco, cigars, etc.)?

- yes
- no

How often do you use one or more of the following tobacco products (cigarettes, snuff, chewing tobacco, cigars)

- never
- monthly
- 2-4 ce per month
- 2 to 3ce a week
- 4 or more times a week

How often do you have a drink containing alcohol? (Check one.)

- never
- monthly
- 2-4 ce per month
- 2 to 3ce a week
- 4ce a week or more

How many drinks containing alcohol do you have on a typical day you choose to drink? (Check one.)

- 1-2
- 3-4
- 5-6
- 7-8
- 9 or more

. What kind of symptoms do you use analgesics (pain medications)? Pick all that apply

- [ ] headaches
- [ ] Body aches/aches
- [ ] stomach aches
- [ ] fever
- [ ] Dysmenorrhoea
- [ ] others, please specify

Please specify

____

which medicine did you last use for pain?

- ◯ Paracetamol
- ◯ Panadol extra or its variants
- ◯ Ibuprofen
- ◯ Diclofenac
- ◯ aspirin (alabukun, phensic)
- ◯ others

Concerning , How much is the maximum number of tablets that can be taken in a day?

- ◯ 1-2
- ◯ 3-4
- ◯ 5-6
- ◯ 7-8
- ◯ others

PLease specify

____

Concerning , How many tablets will you consider to make an effective dose?

- ○ 1
- ○ 2
- ○ 3
- ○ 4
- ○ more than 4

Is there any complication associated with the excessive use of ?

- ○ yes
- ○ no
- ○ don't know

if yes, check all that apply

- ☐ nausea and vomiting
- ☐ kidney damage
- ☐ Liver damage
- ☐ Peptic ulcer
- ☐ heart burn
- ☐ others specify

Please specify

____

How many tablets make an effective dose of paracetamol?

- ○ 1-2
- ○ 3-4
- ○ more than 4

Is there any complication associated with an excessive use of paracetamol

- ○ yes
- ○ no
- ○ don't know

If yes, tick all that apply

- nausea and vomitting
- kidney damage
- liver damage
- other specify

Please specify

Have you in the past self-medicated with antibiotics?

- yes
- no

Have you read the insert leaflet of any antibiotics?

- yes
- no

Have you self-medicated with antibiotics in the last 6 months

- yes
- no

If yes to the above, how many times in the last 6 months

- 1
- 2
- 3
- 4
- more than 4

Which did you use, if yes

- [ ] ampicillin
- [ ] . ampiclox
- [ ] amoxicilin
- [ ] clavulanate+amoxicillin
- [ ] ciprofloxacin
- [ ] tetracycline
- [ ] others to specify

Please specify

---

concerning ampiclox, how many capsules makes an effective dose for an adult?

- ○ 1
- ○ 2
- ○ 3
- ○ 4
- ○ more than 4

. concerning ampiclox, how many times its to be taken in a day for an adult?

- ○ 1
- ○ 2
- ○ 3
- ○ 4
- ○ more than 4

Is there any complication associated with the excessive intake of ampiclox?

- ○ yes
- ○ no

If yes, Check all that apply

- nausea and vomiting
- diarrhoea
- rashes
- anaphylaxis/collapse
- others

Please specify

HEALTH LITERACY

HEALTH LITERACY

How often are appointment slips written in a way that is easy to read and understand?

- Always
- Often
- Sometimes
- Occasionally
- Never

How often are medical forms written in a way that is easy to read and understand?

- Always
- Often
- Sometimes
- Occasionally
- Never

How often are medication labels written in a way that is easy to read and understand?

- Always
- Often
- Sometimes
- Occasionally
- Never

How often are patient educational materials written in a way that is easy to read and understand?

- Always
- Often
- Sometimes
- Occasionally
- Never

How often are hospital or clinic signs difficult to understand?

- Always
- Often
- Sometimes
- Occasionally
- Never

How often are appointment slips written in a way that is easy to read and understand?

- Always
- Often
- Sometimes
- Occasionally
- Never

How often do you have problems completing medical forms because of difficulty understanding the instructions?

- Always
- Often
- Sometimes
- Occasionally
- Never

How often are appointment slips difficult to understand?

- Always
- Often
- Sometimes
- Occasionally
- Never

How often are medical forms difficult to understand and fill out?

- ○ Always
- ○ Often
- ○ Sometimes
- ○ Occasionally
- ○ Never

How often are directions on medication bottles difficult to understand?

- ○ Always
- ○ Often
- ○ Sometimes
- ○ Occasionally
- ○ Never

How often do you have problems learning about your medical condition because of difficulty understanding written information?

- ○ Always
- ○ Often
- ○ Sometimes
- ○ Occasionally
- ○ Never

How often do you have difficulty understand written information your health care provider (like a doctor, nurse, nurse practitioner) gives you?

- ○ Always
- ○ Often
- ○ Sometimes
- ○ Occasionally
- ○ Never

How often do you have problems getting to your clinic appointments at the right time because of difficulty understanding written instructions?

- Always
- Often
- Sometimes
- Occasionally
- Never

How often are you unsure of how to take your medication(s) correctly because of problems understanding written instructions on the bottle label?

- Always
- Often
- Sometimes
- Occasionally
- Never

How confident are you filling out medical forms by yourself?

- Always
- Often
- Sometimes
- Occasionally
- Never

How confident do you feel you are able to follow the instructions on the label of a medication bottle?

- Always
- Often
- Sometimes
- Occasionally
- Never

. How confident are you filling out medical forms by yourself?

- Always
- Often
- Sometimes
- Occasionally
- Never

BRIEF

BRIEF: Health Literacy Screening Tool Please circle the answer that best represents your response.

1. How often do you have someone help you read hospital materials?

- Always
- Often
- Sometimes
- Occasionally
- Never

2. How often do you have problems learning about your medical condition because of difficulty understanding written information?

- Always
- Often
- Sometimes
- Occasionally
- Never

3. How often do you have a problem understanding what is told to you about your medical condition?

- Always
- Often
- Sometimes
- Occasionally
- Never

4. How confident are you filling out medical forms by yourself?

- ◯ Always
- ◯ Often
- ◯ Sometimes
- ◯ Occasionally
- ◯ Never
